# The cyclic nucleotide gated cation channel AtCNGC10 traffics from the ER via Golgi vesicles to the plasma membrane of Arabidopsis root and leaf cells

**DOI:** 10.1186/1471-2229-7-48

**Published:** 2007-09-19

**Authors:** David A Christopher, Tamas Borsics, Christen YL Yuen, Wendy Ullmer, Christine Andème-Ondzighi, Marilou A Andres, Byung-Ho Kang, L Andrew Staehelin

**Affiliations:** 1 University of Hawaii, Dept. of Molecular Biosciences & Bioengineering, 1955 East-West Rd. Honolulu, HI 96822, USA; 2 University of Colorado at Boulder, Molecular, Cellular & Developmental Biology, UCB 347 Boulder, CO 80309-0347, USA; 3 University of Hawaii, Pacific Biosciences Research Center, Honolulu, HI 96822, USA

## Abstract

**Background:**

The cyclic nucleotide-gated ion channels (CNGCs) maintain cation homeostasis essential for a wide range of physiological processes in plant cells. However, the precise subcellular locations and trafficking of these membrane proteins are poorly understood. This is further complicated by a general deficiency of information about targeting pathways of membrane proteins in plants. To investigate CNGC trafficking and localization, we have measured *Atcngc5 *and *Atcngc10 *expression in roots and leaves, analyzed AtCNGC10-GFP fusions transiently expressed in protoplasts, and conducted immunofluorescence labeling of protoplasts and immunoelectron microscopic analysis of high pressure frozen leaves and roots.

**Results:**

AtCNGC10 mRNA and protein levels were 2.5-fold higher in roots than leaves, while AtCNGC5 mRNA and protein levels were nearly equal in these tissues. The AtCNGC10-EGFP fusion was targeted to the plasma membrane in leaf protoplasts, and lightly labeled several intracellular structures. Immunofluorescence microscopy with affinity purified CNGC-specific antisera indicated that AtCNGC5 and AtCNGC10 are present in the plasma membrane of protoplasts. Immunoelectron microscopy demonstrated that AtCNGC10 was associated with the plasma membrane of mesophyll, palisade parenchyma and epidermal cells of leaves, and the meristem, columella and cap cells of roots. AtCNCG10 was also observed in the endoplasmic reticulum and Golgi cisternae and vesicles of 50–150 nm in size. Patch clamp assays of an AtCNGC10-GFP fusion expressed in HEK293 cells measured significant cation currents.

**Conclusion:**

AtCNGC5 and AtCNGC10 are plasma membrane proteins. We postulate that AtCNGC10 traffics from the endoplasmic reticulum via the Golgi apparatus and associated vesicles to the plasma membrane. The presence of the cation channel, AtCNGC10, in root cap meristem cells, cell plate, and gravity-sensing columella cells, combined with the previously reported antisense phenotypes of decreased gravitropic and cell enlargement responses, suggest roles of AtCNGC10 in modulating cation balance required for root gravitropism, cell division and growth.

## Background

Cations are essential macro- and micronutrients in plants, playing critical roles in many cellular processes, such as signal transduction, disease resistance, osmotic equilibrium, growth, and development [reviewed in [1,2]]. Although required for normal cellular functions, an imbalance of cations can cause deleterious effects in plants. To regulate intracellular cation homeostasis, plants have evolved several distinct classes of transporters to facilitate the movement of monovalent and divalent cations across cellular membranes. One such class of cation transporters is the cyclic nucleotide-gated ion channels (CNGCs) [3].

**Figure 1:**
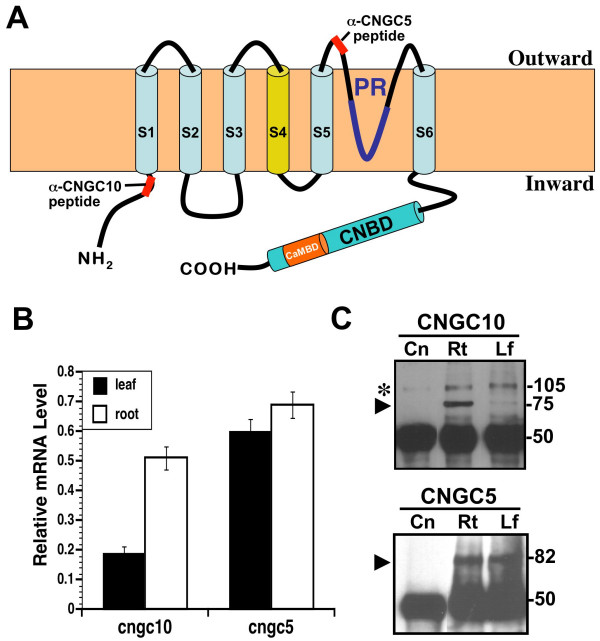
**Structure and expression of CNGCs in Arabidopsis**. **(A) **Two dimensional model of a generic CNGC as positioned in the membrane relative to the inward and outward orientations. Locations of the epitopes for the AtCNGC10- and AtCNGC5-specific antisera are shown. Six transmembrane domains (S1-S6), the pore (PR), cyclic nucleotide-binding (CNBD) and calmodulin-binding domains (CaMBD) are indicated. **(B) **Quantitation of relative mRNA levels for the *Atcngc10 *and *Atcngc5 *genes using quantitative RT-PCR. *Cngc *mRNA levels were normalized to actin mRNA levels. **(C) **Immunoprecipitation of AtCNGC10 and AtCNGC5 from root (Rt) and leaf (Lf) total cellular proteins vs. a negative control (Cn, *E. coli *total protein). Molecular sizes of protein bands are indicated in kilodaltons (kDa). The black arrowhead denotes the band corresponding to each CNGC. The asterisk (*) refers to a non-specific band detected in all samples.

Originally discovered in barley [4] and subsequently found in many plant species [5], CNGCs share a Shaker-like structure composed of six membrane-spanning domains (S1-6) with a pore situated between S5 and S6 (Figure 1A) [3,6]. The hydrophilic C-terminus contains partially overlapping cyclic nucleotide-binding (CNBD) and calmodulin-bind (CaMBD) domains [7,8]. Cyclic nucleotides (cAMP or cGMP) are believed to activate, whereas calmodulin inhibits cation transport [9-13]. The CNGC family in Arabidopsis is comprised of 20 members [3,14]. Electrophysiological, molecular and genetic complementation studies of six of these members indicate that they are generally non-selective, monovalent and divalent cation channels [13-17], although some discrimination between cations has also been observed [10,11,13,18]. The phenotypic characterization of loss-of-function mutants has implicated CNGCs to be involved in a wide range of plant processes [14] including tolerance to heavy metals [7,19], sensitivity to various cation stresses [17,20], plant development and germination [17,20], programmed cell death, and disease resistance [11,21].

Studies on CNGC localization have lagged behind the biochemical, electrophysiological and genetic investigations of CNGC activity. Cell disruption and fractionation experiments indicate that the tobacco homolog, NtCBP4, is associated with the plasma membrane [7]. When transiently expressed, AtCNGC3-GFP fusion proteins were detected in the outer region of the leaf protoplast [17], also suggesting that AtCNCG3 is targeted to the plasma membrane. However, no CNGCs have been immunolocalized at the electron microscope level of analysis and no data exists on their intracellular trafficking in plants. The latter point is also important because the pathway(s) by which membrane proteins are transported to the plasma membrane in plants are not fully understood [22].

To determine where different CNGCs function in plant cells and to elucidate their intracellular trafficking mechanism, we have analyzed the expression of AtCNGC5 and AtCNGC10 and determined at high spatial-resolution the subcellular localization and trafficking of AtCNGC10. AtCNGC10 plays an important role in numerous growth responses in plants [23]. It also modulates potassium levels in tissues, and it can rescue potassium uptake mutants of *E. coli*, yeast and Arabidopsis on low potassium media [13]. In this report, combined approaches of AtCNGC10-GFP targeting and immunofluorescence and electron microscopy of the native protein were used to show that AtCNGC10 is present in the plasma membrane of a variety of cell types in roots and leaves. We have also localized AtCNGC10 to the endoplasmic reticulum cisternae, Golgi stacks and vesicles, and vesicles near the plasma membrane, consistent with the notion that this transporter is synthesized in the ER and transported via the Golgi to the plasma membrane. Finally, patch clamp assays of HEK293 cells heterologously expressing the AtCNGC10-GFP fusion indicated that the AtCNGC10 channel transported K^+^.

## Results and discussion

To study the location of CNGCs within the cellular membranes of Arabidopsis, we chose a combination of CNGC-GFP targeting and immunological approaches. Affinity purified, AtCNGC5- and AtCNGC10-specific polyclonal antisera were generated against synthetic peptides derived from the unique sequences of less conserved sub-regions within the CNGC family. The corresponding positions of these peptides within the predicted two-dimensional structure of a representative generic CNGC are shown (Figure 1A). The AtCNGC10-specific antiserum binds to the hydrophilic N-terminus just upstream from the first transmembrane domain, while the AtCNGC5 antiserum binds to an exposed outer loop between the fifth transmembrane domain and the pore (Figure 1A). The specificity of the AtCNGC10-specific antiserum has been characterized in detail against recombinant AtCNGC10 and native plant proteins on immunoblots [23]. The specificity of the AtCNGC5 antiserum was determined here (Additional File 1).

The expression levels of *Atcngc5 *and *Atcngc10 *were assessed in mature leaves and roots using both real-time RT-PCR (Figure 1B) and immunoprecipitation of total cellular proteins (Figure 1C). The abundance of *Atcngc5 *mRNA was similar in roots and leaves, whereas *Atcngc10 *mRNA levels were over 2.5-fold greater in roots. In addition, *Atcngc5 *mRNA levels were 3-fold higher than that of *Atcngc10 *in leaves and 20% higher in roots. In our immunoprecipitation experiments, the AtCNGC10 and AtCNGC5 antisera detected specific bands at 75 and 82 kDa, respectively (Figure 1C), which corresponded to the predicted sizes of the deduced polypeptides. Consistent with our real time RT-PCR results, AtCNGC5 was more abundant than AtCNGC10 in both tissues. Generally, CNGC protein levels correlated with mRNA levels on an equal protein loading basis and using equal antibody dosages (50 kDa heavy chain, Figure 1C) in the immunoprecipitation assay.

**Figure 2:**
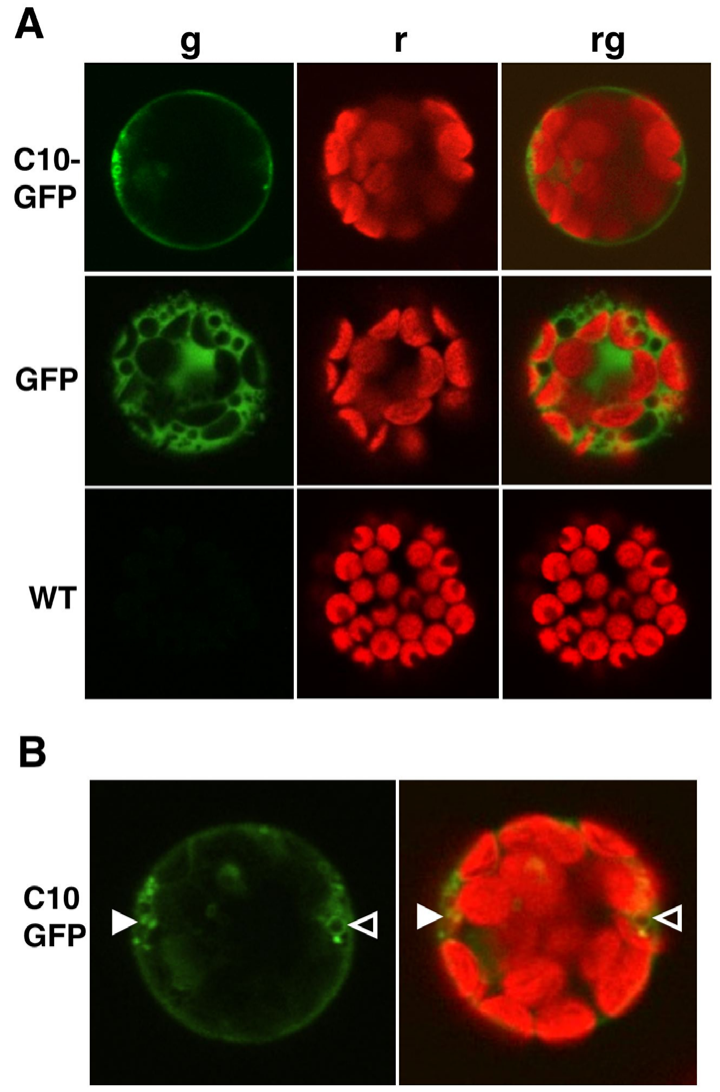
**Transient expression reveals AtCNGC10-GFP trafficking to the plasma membrane of Arabidopsis leaf protoplasts using confocal laser scanning microscopy**. **(A) **Leaf protoplasts were transfected with the two constructs pBL-35S:AtCNGC10-EGFP (C10-GFP) and pBL-35S:EGFP alone (GFP), and compared with untransfected controls (WT). Images were acquired through laser scanning confocal microscopy and include: (**g**) a 505–525 nm emission filter for GFP, (**r**) chlorophyll autofluorescence detected using a 650 nm emission filter, and (**rg**) merged GFP and chlorophyll autofluorescence. **(B) **Solid white arrowheads label AtCNGC10-GFP in punctate regions, while open arrowheads label vesicle-like structures.

As an initial approach for localizing CNGCs within the cell, we employed transient expression of an AtCNGC10-GFP fusion controlled by the 35S CaMV promoter in Arabidopsis leaf protoplasts (Figure 2). The AtCNGC10 sequence directed GFP to the plasma membrane, whereas the GFP-alone control was detected uniformly in the cytosol (Figure 2A). The AtCNGC10-GFP construct also lightly labeled some diffuse as well as several distinct punctate intracellular structures (Figure 2B). These results suggested that the AtCNGC10 sequence directed the fused GFP through the secretory pathway to the plasma membrane. In another study, some similar structures were also reported to be labeled with AtCNGC3-GFP [17].

**Figure 3:**
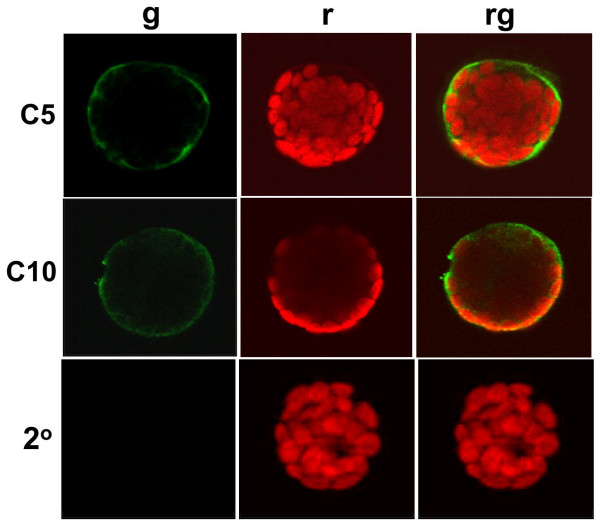
**Immunolocalization of AtCNGC5 and AtCNGC10 to the plasma membrane of Arabidopsis leaf protoplasts using confocal laser scanning microscopy**. Wild type leaf protoplasts were challenged with primary antibodies specific to AtCNGC5 (C5), and AtCNGC10 (C10), followed by AlexaFluor488 anti-rabbit secondary antibodies. A negative control using only the secondary antibody was also performed (2°). Exposures included (**g**) a 505–525 nm emission filter for AlexaFluor488, (**r**) chlorophyll autofluorescence detected using a 650 nm emission filter, or (**rg) **both immunofluorescence and chlorophyll autofluorescence merged.

To confirm the results obtained with the AtCNGC10-GFP fusion construct, we conducted immunolocalization experiments to determine the localization patterns of native CNGC proteins in cells. Immunolabeling at the light microscopy level was performed on chemically fixed wild type leaf protoplasts using the anti-AtCNGC5 and anti-AtCNGC10 antisera (Figure 3) and an Alexafluor488-conjugated anti-rabbit secondary antiserum. Fluorescence was specifically detected around the outer fringe of the protoplast coincident with the plasma membrane (Figure 3). However, to obtain a strong signal with the AtCNGC10 required 30 min longer incubations with the anti-AtCNGC10 antiserum than with the anti-AtCNGC5 antiserum, possibly to allow for the antisera to penetrate the protoplast to access the epitope on the cytosolic side of the membrane. The negative controls consisted of incubation of fixed protoplasts with the Alexaflour488-conjugated anti-rabbit secondary antibody alone, which produced no signal, indicating that the immunolocalizations of anti-AtCNGC5 and AtCNGC10 to the plasma membrane resulted from primary antibody localization and not background binding of the secondary antibody.

**Figure 4:**
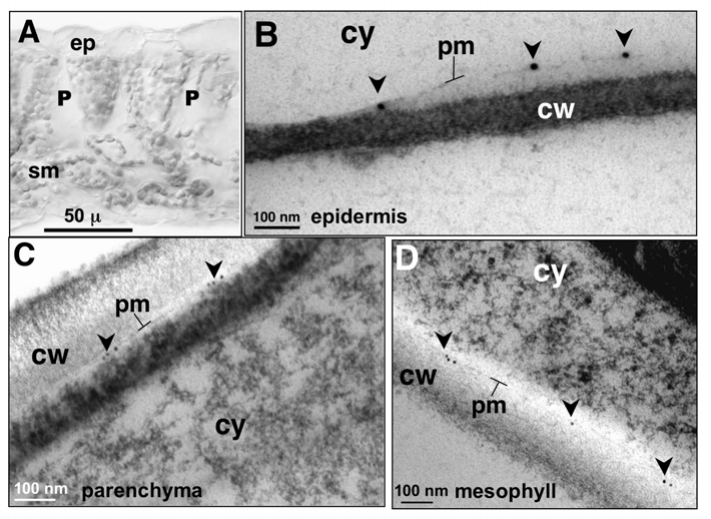
**Immunolocalization of AtCNGC10 to the plasma membrane of diverse Arabidopsis leaf cells using transmission electron microscopy**. (**A**) Bright field cross-section view of leaf cells examined, ep, epidermis; P, palisade parenchyma; sm, spongy mesophyll. (**B**) Partially plasmolyzed epidermal cell labeled with anti-AtCNGC10 antiserum and 15 nm gold secondary antibody on plasma membrane (pm, arrows). Plasma membranes of palisade parenchyma cell (**C**) and spongy mesophyll cell **(D) **labeled with anti-AtCNGC10 antiserum and 10 nm gold secondary antibody (arrows). Cell wall, cw, and cytoplasm, cy, are denoted in each panel. Size bars are in microns (μ) or nanometers (nm). Controls with secondary antisera alone gave no signal (data not shown).

The evidence of AtCNGC10-GFP targeting (Figure 2) and immunofluorescence localization of both AtCNGC5 and AtCNGC10 to the plasma membrane in protoplasts (Figure 3) prompted us to conduct further experiments using electron microscopy of high pressure frozen leaves to obtain higher resolution immunolocalization data (Figure 4). Unfortunately, the AtCNGC5 antiserum proved problematic on high pressure frozen and acetone-substituted thin tissue sections. However, the AtCNGC10 antiserum gave very specific and clean labeling of the plasma membrane of epidermal (Figure 4B), palisade parenchyma (Figure 4C) and spongy mesophyll cells (Figure 4D). Slight plasmolysis of the epidermal cells (Figure 4B) resulted in an increased separation of the plasma membrane from the cell wall, which further highlighted the specificity of immunolabeling in the plasma membrane.

**Figure 5:**
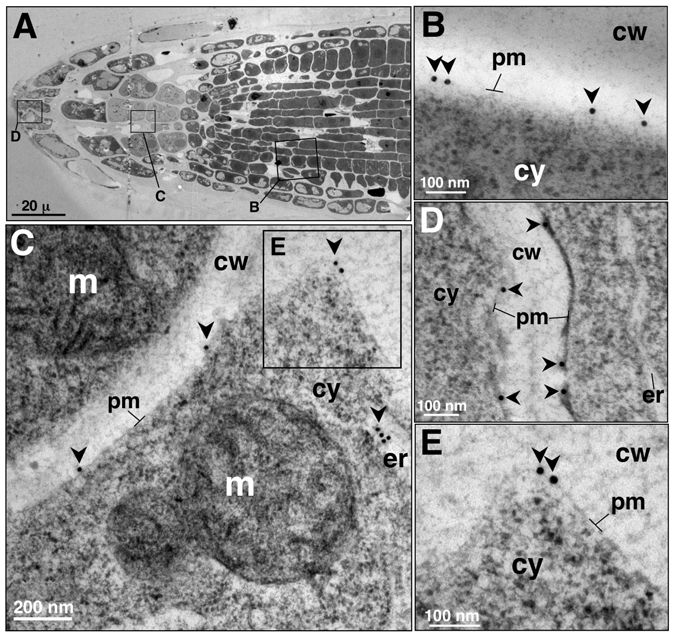
**Immunolocalization of AtCNGC10 to the plasma membrane of Arabidopsis root cells using TEM**. (**A**) Transverse section of root tip showing three regions (rectangles) examined under TEM labeled with anti-AtCNGC10 antiserum. **(B) **Root meristematic cell plasma membrane; **(C) **Root columella cell; m, mitochondria; er, endoplasmic reticulum. **(D) **border tip cell. **(E) **Immunolabeled region in the box from panel C under higher magnification. 15 nm gold anti-rabbit secondary antibody used in all labeling. Cell wall, cw, and cytoplasm, cy. Size bars are in nanometers (nm).

AtCNGC10 was also detected in the plasma membrane of various cell types within the root tip (Figure 5A). Labeling was especially high in the cells of the meristematic zone (Figure 5B), columella cells (Figure 5C) and the root cap (Figure 5D). The detection of AtCNGC10 in the plasma membrane of the gravity-sensing columella cells was especially interesting. We have previously shown that roots from the antisense lines of *Atcngc10 *are impaired in their response to gravity compared to wild type controls [23]. We suggest that the effects of AtCNGC10 on root gravity response could be mediated by cation transport across the plasma membrane of columella cells.

**Figure 6:**
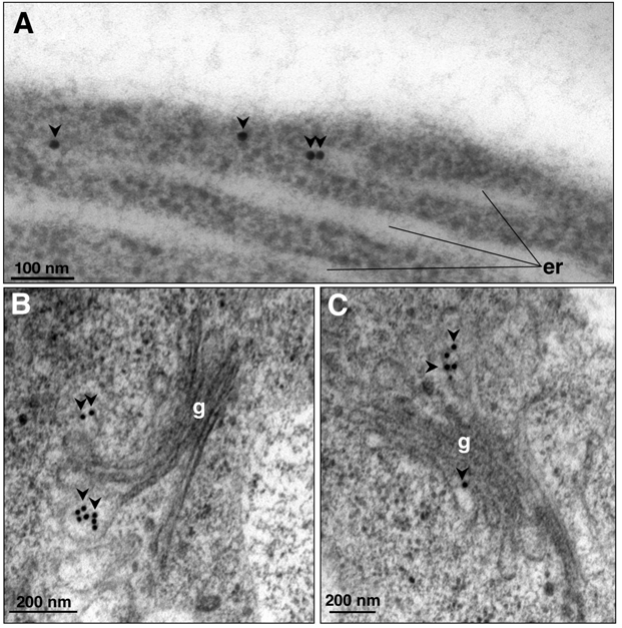
**Immunolocalization of AtCNGC10 to the endoplasmic reticulum and Golgi vesicles of Arabidopsis root cells using transmission electron microscopy**. (**A**) Endoplasmic reticulum (er); **(B) **and **(C) **Golgi apparatus (g) labeled with anti-AtCNGC10 antiserum. Arrows denote 15 nm immunogold particles of anti-rabbit secondary antiserum. Size bars are in nanometers (nm).

**Figure 7:**
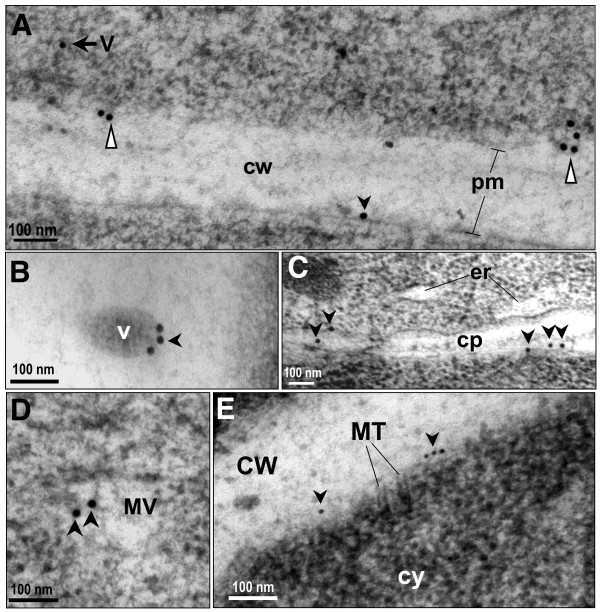
**Characterization of AtCNGC10 associated with vesicles and cell plate**. (**A**) Immunogold labeling of cytoplasmic vesicle (V, black arrow) and vesicles merging with plasma membrane, pm (white triangles). Black arrowhead indicates 15 nm immunogold in pm. (**B**) Three immunogold particles from anti-AtCNGC10 antiserum labeling on edge of vesicle (V); (**C**) Cell plate (cp) formation with anti-AtCNGC10 antiserum labeling indicated with black arrowheads. Endoplasmic reticulum (er). (**D**) Multiple vesicles present in cytoplasm labeled with anti-AtCNGC10 antiserum; (**E**) Anti-AtCNGC10 antibody labeling near cortical microtubule (MT) attachments. cw, cell wall. Size bars are in nanometers (nm).

To identify all of the intracellular structures containing AtCNGC10, we have further analyzed immunogold labeled thin sections of high pressure frozen root tip cells using the anti-AtCNGC10 antibody. Immunogold particles were observed over ER cisternae (Figures 5C, 6A), Golgi stacks and emerging trans-Golgi vesicles (Figures 6B, C), secretory vesicles (Figures 7A, 7B, 7D) and vesicles merging with the plasma membrane (Figure 7A). This labeling pattern is consistent with the hypothesis that AtCNGC10 is synthesized by ER-bound ribosomes, passaged through the Golgi and transported in secretory vesicles to the plasma membrane, which is a pathway that is postulated to be followed by most plasma membrane proteins in plants [22]. In addition, anti-AtCNGC10 labeling was also observed in the plasma membrane over late stage cell plates in dividing cells (Figure 7C) and near cortical microtubule attachment sites (Figure 7E). The delivery of AtCNGC10 to cell plates in the state of formation suggests, furthermore, that this ion transporter is of fundamental importance to plasma membrane function in dividing root tip cells.

**Figure 8:**
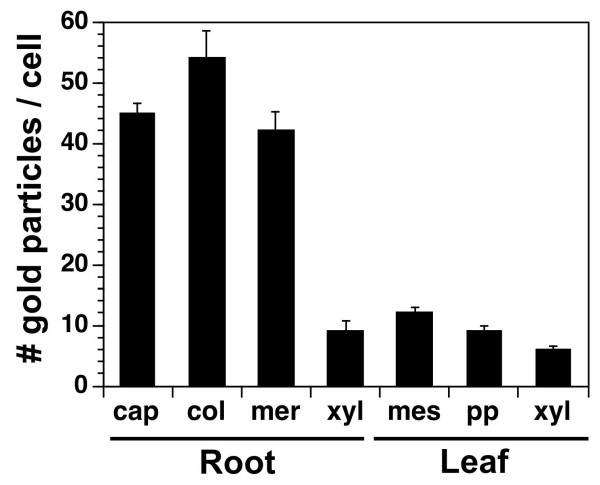
**Quantitation of labeling of root and leaf cells with the anti-AtCNGC10 antiserum**. The number of immuno-gold particles from the anti-rabbit secondary antibody were counted for each cell type, which included root cap, columella (col), meristem (mer) and xylem (xyl), leaf spongy mesophyll (mes) and palisade parenchyma (pp). Data represents average immunogold particles from 10 cells per experiment (total two experiments) ± standard error.

The overall labeling of the anti-AtCNGC10 antibody over leaf and root cells roots was quantitated on a per cell basis (Figure 8). Labeling was four-fold higher in cap, meristem and columella cells of roots than xylem cells of roots. This suggests that AtCNGC10 was not a major channel for cation loading of xylem cells in roots. Likewise, labeling over leaves was on average one-fourth the level of labeling in root tip cells. The higher abundance of AtCNGC10 labeling in roots relative to leaves was in agreement with the expression analysis (Figure 1B, 1C), and suggests that AtCNGC10 plays a more predominant role in roots.

**Figure 9:**
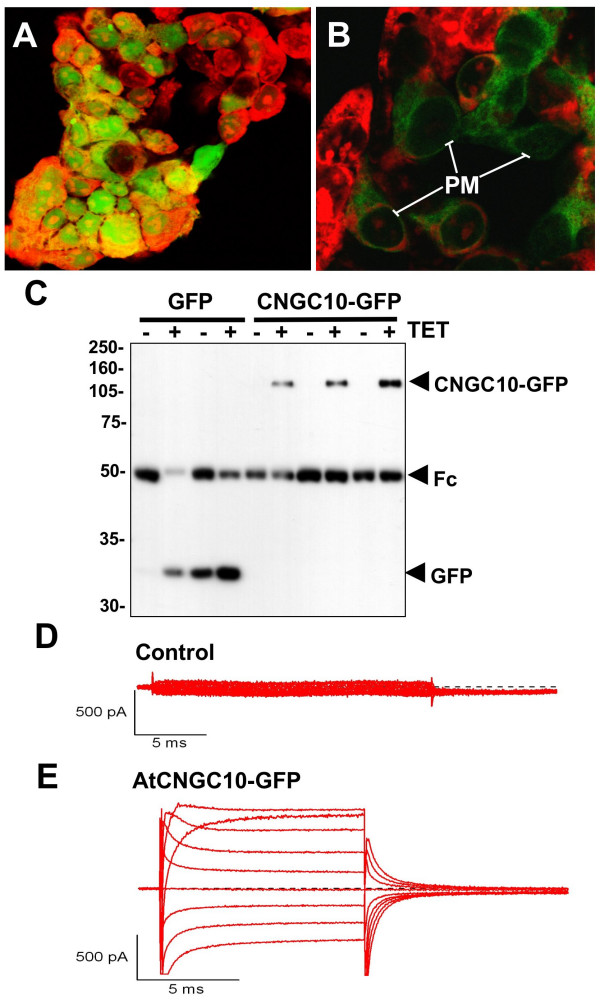
**Expression of AtCNGC10-GFP in HEK293 cells and patch clamp assays**. Transient expression of **(A) **GFP alone in the vector and **(B) **the AtCNGC10-GFP fusion in HEK293 cells and visualized under the fluorescence microscope. HEK cells were fixed with 4% dimethyl-formamide and stained with propidium iodide (red fluorescence). Distinct GFP labeling of peripheral plasma membrane (PM) is marked. Immunoprecipitation of the cells expressing each construct with and without the inducer tetracycline (+/-) TET is shown. The bands corresponding to the GFP alone and AtCNGC10-GFP fusion are labeled. The Fc region of the antiserum used in immunoprecipitation is marked. Proteins were detected on immunoblots with the anti-FLAG antibody. **((D) **Non-transfected HEK cell showed no measurable current. **(E) **HEK cell transfected with AtCNGC10-GFP showed large inward and outward currents in asymmetrical solution consisting of 145 K^+^_out_/145 NMDG_in_. Shown are leak-subtracted currents generated from command voltages between -60 to +100 mV in the presence of 100 μM dibutyryl cGMP.

To test for the ability of AtCNGC10 to mediate K^+ ^transport in an independent heterologous system, we transiently expressed AtCNGC10-GFP in HEK293 cells (Figure 9). AtCNGC10-GFP distinctly labels the periphery and some endomembrane structures of the HEK293 cell, whereas GFP alone labels the cell somewhat uniformly. Immunoprecipitation assays indicated that the two constructs were expressed and that AtCNGC10 was fused to GFP based on the predicted size on SDS-PAGE. In patch clamp assays, we observed that heterologously expressed AtCNGC10 conducted significant inward K^+ ^currents with the application of dibutyryl cGMP (100 uM) in the extracellular recording solution, demonstrating the activation of these channels [5]. We also observed significant outward currents, which may be carried by N-methyl-D-glucamine, a large organic cation, through the AtCNGC10. Further work is therefore needed to characterize the selectivity of AtCNGC10 for small and large cations and the role of calmodulin and cyclic nucleotides in channel regulation.

The ability of *Atcngc10 *to rescue *E. coli *and yeast mutants defective in K^+ ^transport [13], combined with the plasma membrane localization of AtCNGC10 in a variety of cells and cation transport in heterologous HEK293 cells shown here, is consistent with its role in regulating cellular expansion through K^+ ^transport [23] via the acid growth mechanisms [24-29]. Antisense knockdown lines of *Atcngc10 *had smaller parenchyma cells and leaves and were disrupted in rates of root growth and gravitropic bending [23]. Therefore, it is reasonable to propose that AtCNCG10 is involved in cation uptake in the plasma membrane of root and leaf cells. In addition, AtCNGC10 may also be involved in cation transport across the membrane of the vesicles in which it was detected. The inability to isolate a true knockout of AtCNGC10 and the observed haploinsufficiency [23], combined with the cell biological data observed here, further support our contention that AtCNGC10 is an essential gene in plants.

## Conclusion

AtCNGC5 is equally expressed in roots and leaves, while AtCNGC10 is expressed preferentially in roots. Both channels were localized to the plasma membrane of leaf protoplasts using immunofluorescence microscopy, and the translational fusion between AtCNGC10 and GFP also localized to the plasma membrane, indicating that it targets through the secretory pathway. High-resolution immunogold labeling at the EM level demonstrated that AtCNGC10 resides in the plasma membrane of root tip, columella, and meristematic cells, as well as in leaf epidermal, mesophyll and palisade parenchyma cells. AtCNGC10 was found in the ER, Golgi and vesicles, and we propose that these vesicles are trafficking intermediates in the secretory pathway for plasma membrane proteins. AtCNGC10 most likely transports cations into actively growing cells of leaves and roots and the gravity-sensing columella cells of roots.

## Methods

### Plant material and growth conditions

Experiments were performed using wild type *Arabidopsis thaliana*, Columbia ecotype. Seeds were surfaced sterilized in 70% ethanol for two min, rinsed with sterile water, and suspended in 20% household bleach-0.4% Tween 20 for 5 min, followed by several rinses with sterile water. Seeds were plated on 0.5× MS agar plates and stored at 4°C in the dark for 1–3 days, then exposed to a 14/10 hour light/dark cycle at 23°C for 21–28 days before harvesting for protoplast preparation, and RNA and protein isolation. Plants for electron microscopy were germinated directly on superfine germination medium (Farfard, Inc. Anderson, SC) and exposed to a 14/10 hour light/dark cycle at 25°C for 21–28 days before harvesting for cryofixation.

### RNA isolation and quantitative RT-PCR

Primers for quantitative RT-PCR analysis were generated based on the cDNA sequences of *Atcngc10 *[GenBank:AF272002; [13]] and *Atcngc5 *[GenBank:NM203224]. The primer sequences were designed to span gene exon/exon boundaries (which eliminates contaminating genomic DNA signals) and were checked against the *Arabidopsis *database to ensure their gene specificity: *Atcngc10*: 5'-CCTCGATCTCCTCAAGAAAGTACCT-3' and 5'-TCTCCTTCACGGATCACGTAACT-3'; *Atcngc5*: 5'-ATCAAGCGGCATCTCTGTCT-3' and 5'-CTTTGTGTTCCGGTTCCTGT-3';actin2: 5'-CTTCCGCTCTTTCTTTCCAAGCTC-3' and 5'-ATCATCTCCTGCAAATCCAGCCTTC-3'; Total cellular RNA was isolated as described [30]. Reverse Transcriptions were carried out in 25 ul using 2 ug total RNA and MMLV enzyme (Promega, Co.) according to the users manual. RT-PCR reactions were performed using a SYBR Green PCR Master Mix kit (Applied Biosystems) on a BioRad iCycler Thermal Cycler machine with iCycler Optical Module.

### Antisera, protein isolation, immunoprecipitation and immunoblot analysis

A polyclonal antibody was generated and affinity-purified by New England Peptide, Inc. (Boston, MA) against a predicted antigenic region of AtCNGC5 with the sequence (Ac-NRAKESVLKSKC-amide). The purified antibody was tested for specificity to its peptide and recombinant polypeptide by immunoblotting (data supplement). The generation and testing of the polyclonal antibody specific to AtCNGC10 were described previously [23]. The locations of the epitopic peptides relative to the CNGC polypeptide are shown (Figure 1).

Proteins were extracted from whole 21–28 day-old plants by grinding in liquid nitrogen to a fine powder, then adding 4 vol (w/v) of 1× lysis buffer (50 mM HEPES-pH 7.4, 75 mM NaCl, 40 mM NaF, 10 mM Iodocetamide, 1% IGEPAL, 0.25 mM PMSF, 1 mM Na-orthoVanadate, Calbiochem Protease Inhibitor Cocktail Set IV diluted 1:1000) and mixing well. Samples were kept on ice for 20 minutes with occasional shaking, then centrifuged at 4,000 × g for 20 min at 4°C. The supernatant was removed and 500 μg protein was used for immunoprecipitation. Two μg of anti-ATCNGC5 or AtCNGC10 antibody was added to 500 μl of protein extract and the mixture was rotated at 20 rpm on an orbital shaker at 4°C for 2 hr. 15 μl of Protein-A-Agarose beads (Invitrogen) were added and the mixture was rotated 20 rpm for 45 minutes at 4°C. The agarose beads were centrifuged at 9500 × g for 30 sec at 4°C, and washed three times with 1 ml 1× lysis buffer. Traces of buffer were removed from the pellet and it was resuspended in 25 μl of protein loading buffer, heated to 95°C for 8 min, and gently centrifuged for 30 sec at 2000 × g. Supernatants were loaded onto a 7% denaturing polyacrylamide gel for SDS-PAGE. The proteins were transferred to a nitrocellulose membrane and probed with a 1:3000 dilution of anti-AtCNGC5 or anti-AtCNGC10 primary antibody, followed by a 1:3000 dilution of anti-rabbit horseradish peroxidase-conjugated secondary antibody for chemiluminescence detection using the ECL kit (GE Healthcare, Inc.).

### Localization of AtCNGC5 and AtCNGC10 in Arabidopsis protoplasts

Protoplasts were isolated from 3–4 week-old wild-type leaves as described [31,32]. Approximately 0.2–1 g leaf tissue was sliced into thin strips using a clean razor. The tissue was incubated in 0.5 M mannitol in a sterile petri dish for 1 hr, then the mannitol was aspirated and 20 ml of enzyme solution containing 1% cellulase (MP Biomedicals) and 0.25% pectinase (Calbiochem, Inc.) disolved in (0.5 M mannitol, 8 mM CaCl_2_, pH adjusted to 5.5) was added and was incubated O/N in the dark. The protoplasts were harvested by swirling the plate to free cells from the digested leaf tissue, then purified by centrifugation (200 × g, 5 min) and washed twice in W5 solution (0.5 M mannitol, 154 mM NaCl, 125 mM CaCl_2_, 5 mM KCl, 5 mM glucose, pH adjusted to 5.8). The protoplasts were resuspended in 2 ml Mg/Mannitol solution (0.4 M mannitol, 15 mM MgCl_2_, 4 mM MES) and purified using 5 ml of a 21% sucrose cushion centrifuged for 10 min at 300 × g. The purified protoplasts were removed from the upper layer and counted using a hemacytometer before transfection or immunolabeling.

A PCR-amplified copy of the 35S-*mGFP5*-Nos cassette from binary vector pCAMBIA1302 was cloned between the HindIII and SacI restriction sites of pBluescript KS(+), and the *mGFP5 *coding sequence (between the NcoI and BstEII sites) subsequently replaced with either an *EGFP *fragment or a chimeric *CNGC10-EGFP *fragment to generate constructs pBL(35S:*EGFP*) and pBL(35S:*CNGC10-EGFP*), respectively. The 35S-*mGFP*-Nos fragment was amplified using primers 35S/HindIII.F (5'-CAGTTAAGCTTCATGGAGTCAAAGATTCA-3' and Nos/SacI.R (5'-TAGTTGAGCTCCCGATCTAGTAACATAGA-3', while the non-chimeric version of *EGFP *was generated with primers EGFP/NcoI.F (5'-TAGTCCCATGGTGAGCAAGGGCG-AGGA-3') and EGFP/BstEII.R (5'-TACAGGGTCACCTAGCCGAGAGTGATCCCG-3'), and the chimeric version with primers EGFP/NdeI.F (5'-CACCGACTAGTCATATGGTGAGCAAGGGCGAG-3') and EGFP/BstEII.R. To create pBL(35S:*CNGC10-EGFP*), *CNGC10 *was amplified from the genomic DNA of wild-type seedlings (Col-0 ecotype), using primer pair CNGC10/NcoI.F (5'-TTTCCCATGGTTTTGTTTAGGTTCAAAGATGAAG-3') and CNGC10/NdeI.R (5'-GAGACATATGAGGGTCAGTTGTATGATTGGCGGT-3'). The sequence of the reverse primer modifies the stop codon of *CNGC10 *to a His codon (CAT). Ligation of the *Atcngc10 *and *EGFP *fragments via their engineered NdeI restriction sites places the *EGFP *coding sequence in-frame with the last exon of *Atcngc10*. Protoplasts were transfected using PEG as described [32] and incubated at RT under constant darkness in W5 buffer for 18–20 hours prior to examination with an Olympus Fluoview 1000 laser scanning confocal microscope. EGFP was detected by excitation at 488 nm and a 505–525 nm emission filter, while chloroplast autofluorescence was visualized by excitation at 543 nm and a 650 emission filter.

For immunolocalization experiments, protoplasts purified on sucrose cushions (above) were then centrifuged in 15 ml conical tubes at 200 × g for 5 min at 4°C. The protoplasts were resuspended in 5 mls of fixative (3.5% formaldehyde and 1% glutaraldehyde in 1× PBS) and incubated for 2 hr, shaking at 30 rpm. The fixed cells were centrifuged (200 × g, 5 min), and resuspended in 2 ml of PBS. 1 × 10^5 ^fixed cells in 300 μl 1× PBS were incubated with primary antibody (1:200) for 2 hr (30–40 rpm) in a 24-well microplate. The cells were washed in 500 μl 1× PBS for 5 min and 300 μl of 1× PBS was added with secondary antibody (1:100, goat anti-rabbit Alexafluor 488, Molecular Probes, Inc.) and incubated for 1 hr. The cells were washed gently in 500 μl 1× PBS and viewed via confocal microscopy using the settings described above for detection of EGFP and chloroplast autofluorescence. For leaf cross sections, leaf explants were placed in an antifreeze/fixative that contained 4% (w/v) paraformaldehyde, 20% (v/v), dimethyl sulfoxide, 1% (v/v)Tween 20 and 0.05 M sodium cacodylate at pH 7.4. Samples were stored at 5°C prior to cryosectioning at 30 μ in a Reichert-Jung Cryocut 1800 freezing microtome at -20°C and subsequent viewing on an Olympus BX51 microscope.

### Transmission and immuno-electron microscopy

For immunogold electron microscopy, Arabidopsis leaves (3 weeks-old) and root tips (6 day-old) were cryoprotected with 150 mM sucrose, placed into aluminum sample holders, and frozen in a Baltec 010 high pressure freezer (RMC Boeckeler Instruments, Tucson, AZ). The samples were freeze substituted in 0.1% uranyl acetate/0.25% glutaraldehyde in anhydrous acetone for 5 d at -90°C. After slow warming to for -60°C for 3 d, the samples were rinsed in acetone and infiltrated with 25%, 50%, 75% (24 h each), and 100% (4 d) LR-White (EMS, Fort Washington, PA) in acetone, and UV polymerized at 60°C for 3 d or 100% HM20 (4 d) (EMS, Fort Washington, PA) in acetone, and UV polymerized at -60°C for 3 d. Processing of thin sections for immunolabeling was according to [33] with the anti-CNGC10 antibody being applied for 1.5 h at RT, and the anti-rabbit secondary antibody (10 nm and 15 nm gold, Ted Pella, Inc) for 1.5 hr. Controls were performed by omitting the primary antibody and using pre-immune serum. The sections were viewed in a Philips CM10 microscope (Philips, Hillsboro, OR).

### AtCNGC10-GFP expression in HEK293 cells, immunoprecipitation and patch clamp assays

The full-length AtCNGC10 cDNA was fused to EGFP in the HindIII-EcoRI sites of pEGFP-N1 to create pNC10kozG (kanamycin resistance in bacteria and neomicin resistance in HEK cells) and fused to EGFP and the FLAG epitope in the AflII-XhoI sites of pcDNA5-T/O to create pTGC10F (ampicillin resistance in bacteria and hygromicin resistance in HEK cells). HEK293 cells were grown in 10 cm petri dishes to 60% confluence and transfected with 20 μg endotoxin-free plasmid DNA using Lipofectamine 2000 reagent (Invitrogen). HEK293 cells were also grown on coverslips coated with Poly-D-Lysine. Expression of eGFP was visualized 48 hours after transfection. After 48 hr, transfection efficiency was checked, and cells were fixed with 4% dimethyl-formamide, stained with propdium iodide then visualized at 20× and 40× magnification on an Olympus IX70 fluorescence microscope. For immunoprecipitation, cells were harvested and lysed in 1× IP buffer (50 mM HEPES-pH 7.4, 75 mM NaCl, 40 mM NaF, 10 mM Iodocetamide, 1% IGEPAL, 0.25 mM PMSF, 1 mM Na-orthoVanadate, Calbiochem Protease Inhibitor Cocktail Set IV diluted 1:1000) for 30 min on ice. Anti-flag monoclonal antibody (2 μg) (Sigma) was added to the supernatant and gently rotated 2 h at 4°C. Antibody bound protein complexes were pulled down by adding 15 μl pre-washed Protein-A agarose and incubated 45 min at 4°C. The sample was boiled for 8 min and separated via 10% PAGE. The gel was electroblotted onto PVDF membrane, blocked in 1 × PBS-5% nonfat dry milk and incubated with monoclonal FLAG antibody (1:12500) for 1 hr, followed by incubation with anti-mouse antiserum (1:3000), and visualized using an ECL kit (GE Healthcare, Inc.).

Voltage-clamp measurements were conducted in whole-cell configuration using the EPC-9 patch clamp amplifier (HEKA Electronics, Germany) and Pulse (HEKA Elektronik) data acquisition software. Data were then analyzed off-line using PulseFit (HEKA ElektroniK) and IgorPro (WaveMetrics, Lake Oswego, OR, USA) softwares. Borosilicate glass patch pipettes were pulled to a tip size with resistance of 3–4 MOhm. Recording pipettes were filled with a solution consisting of, in mM: 145 N-methyl-D-glucamine, 10 HEPES-H_3_PO_4_, 0.5 MgCl_2_, and pH 7.2. Cells were kept in the bath solution (in mM): 145 KCl, 10 HEPES-KOH, 10 D-glucose, 0.1 MgCl_2_, 100 μM dibutyryl cGMP, and pH 7.4. Currents were generated from command voltages between -160 to +100 mV in 20 mV increments (from and to V_hold _at 0 mV). Linear and capacitive currents were subtracted on line by a P/4 protocol. All experiments were conducted at 25°C.

## Abbreviations

CNGC - Cyclic nucleotide-gated channel.

CaMBD - Calmodulin binding domain.

CNBD - Cyclic nucleotide binding domain.

ER - Endoplasmic reticulum.

HEK293 - Human embryonic kidney cells line 293.

IP - Immunoprecipitation.

TEM - Transmission electron microscopy.

## Authors' contributions

DC conceived the project, did the immunolabeling of tissue specimens, electron microscopy, analyzed all data and wrote the paper. TB analyzed gene expression and did immunopreciptation assays of both plant and HEK293 cells, made AtCNGC10-GFP constructs for HEK293 cell expression and assisted with patch-clamp assays. CY created and assayed the expression of AtCNGC10-GFP fusions in protoplasts. WU did the immunolabeling of protoplasts. MA carried out the patch-clamp experiments. CAO and BHK conducted the high-pressure freezing, substitution, embedding and sectioning of tissues. LAS advised on EM methodology, tissue preparation and data analysis. All authors read and approved the final manuscript.

## Supplementary Material

Additional file 1 Specificity of the AtCNGC5 antiserum. Click here for file
