# Supplementary figures and images for: The cyclic nucleotide gated cation channel AtCNGC10 traffics from the ER via Golgi vesicles to the plasma membrane of Arabidopsis root and leaf cells
Source: BMC Plant Biol. 2007 Sep 19;7:48. doi: 10.1186/1471-2229-7-48 (PMC2031891; doi:10.1186/1471-2229-7-48)

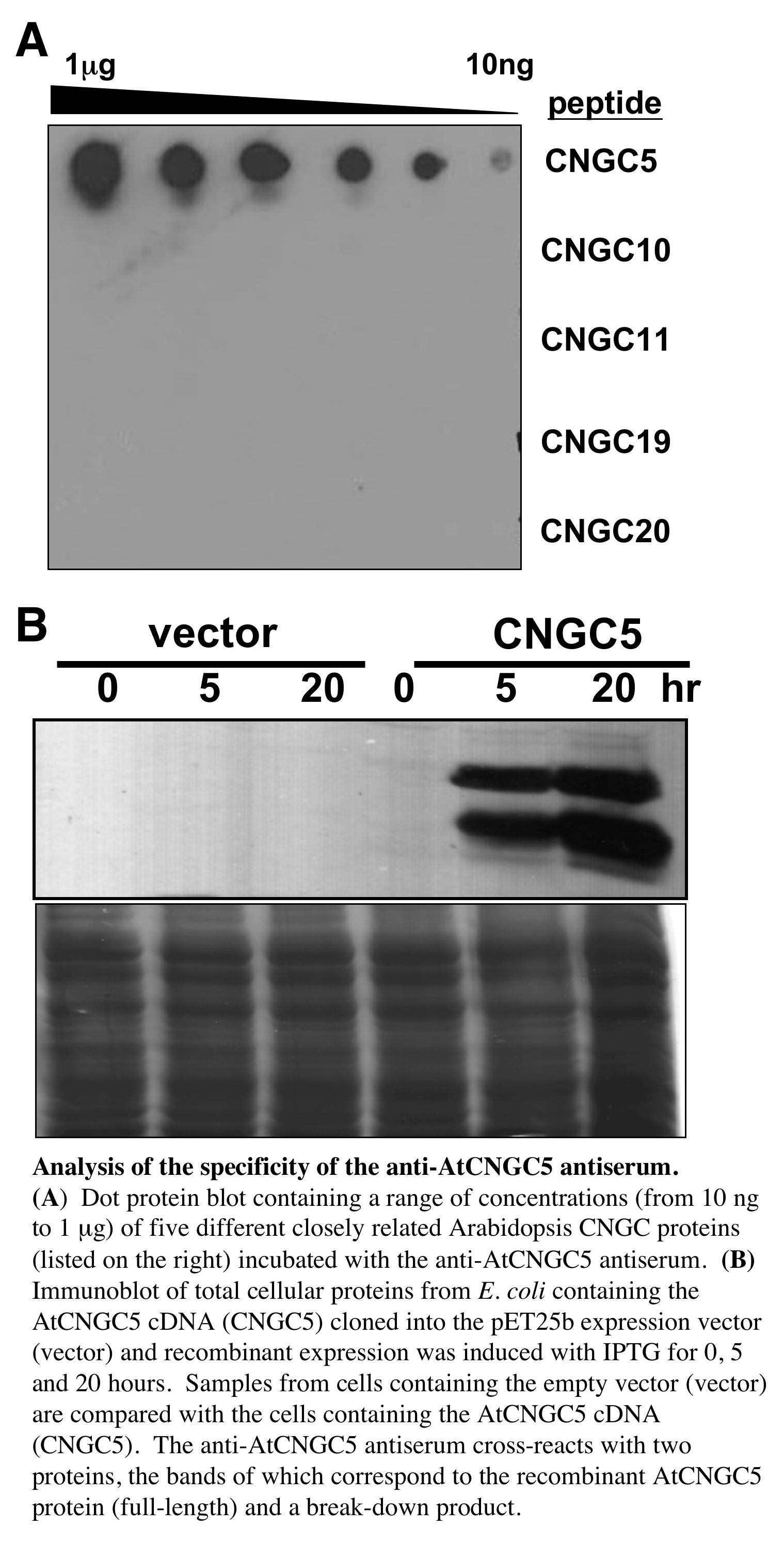

Supplement: Additional file 1 — Specificity of the AtCNGC5 antiserum. [file 1471-2229-7-48-S1.jpeg]
